# Investigation of the Roles of Phosphatidylinositol 4-Phosphate 5-Kinases 7,9 and Wall-Associated Kinases 1–3 in Responses to Indole-3-Carbinol and Biotic Stress in Arabidopsis Thaliana

**DOI:** 10.3390/biom14101253

**Published:** 2024-10-03

**Authors:** Hala Khamesa-Israelov, Alin Finkelstein, Eilon Shani, Daniel A. Chamovitz

**Affiliations:** 1 School of Plant Sciences and Food Security, Tel Aviv University, Ramat Aviv 69978, Israel; kh.hala19@gmail.com (H.K.-I.); alinfin@gmail.com (A.F.); eilonsh@tauex.tau.ac.il (E.S.); 2 Department of Life Sciences, Ben-Gurion University of the Negev, Beer Sheva 84105, Israel

**Keywords:** indole-3-carbinol, glucosinolates, phosphatidylinositol 4-phosphate 5-kinas, Wall-Associated Kinases

## Abstract

Indole-3-carbinol (I3C), a hydrolysis product of indole-3-methylglucosinolate, is toxic to herbivorous insects and pathogens. In mammals, I3C is extensively studied for its properties in cancer prevention and treatment. Produced in Brassicaceae, I3C reversibly inhibits root elongation in a concentration-dependent manner. This inhibition is partially explained by the antagonistic action of I3C on auxin signaling through TIR1. To further elucidate the mode of action of I3C in plants, we have employed a forward-genetic amiRNA screen that circumvents functional redundancy. We identified and characterized two amiRNA lines with impaired I3C response. The first line, *ICT2*, targets the phosphatidylinositol 4-phosphate 5-kinase family (PIP5K), exhibiting tolerance to I3C, while the second line, *ICS1*, targets the Wall-Associated Kinases (WAK1–3) family, showing susceptibility to I3C. Both lines maintain I3C-induced antagonism of auxin signaling, indicating that their phenotypes are due to auxin-independent mechanisms. Transcript profiling experiments reveal that both lines are transcriptionally primed to respond to I3C treatment. Physiological, metabolomic, and transcriptomic analysis reveal that these kinases mediate numerous developmental processes and are involved in abiotic and biotic stress responses.

## 1. Introduction

Glucosinolates (GLSs) are a diverse set of biologically active secondary metabolites found in the Cruciferae family of plants, including broccoli, cabbage, cauliflower, rapeseed, mustard, and the well-known model plant *Arabidopsis thaliana*. GLSs contain nitrogen and acidic sulfate groups and are derived from glucose and an amino acid [1]. Indole glucosinolates are a class of glucosinolates which are most commonly induced during biotic stress, such as in response to fungal pathogens, bacterial elicitors, root flies, caterpillars, flea beetles, and aphids, and following mechanical wounding and treatment with methyl jasmonate [2]. GLSs have several hundred possible breakdown products. These breakdown products, in addition to their bioactive properties, cause the characteristic sharp taste of cruciferous vegetables [3]. These breakdown products typically have either a direct deterrent effect on generalist herbivores or an indirect function as attractants to predatory and parasitic insects. In addition to their toxic effects on herbivorous insects, GSL breakdown products may also signal further plant defense responses [4].

After a tissue rupture event occurs, myrosinase enzymes cleave the most abundant glucosinolate, Indol-3-ylmethylglucosinolat (I3M), producing unstable thiohydroxamate-O-sulfonates, which can later be broken down further to produce different insect-deterrent compounds. Among these is indole-3-carbinol (I3C), which is rapidly produced from Indolylmethylisothiocyanate. I3C regulates auxin response by antagonizing auxin binding to the Transport Inhibitor Response (TIR1) receptor [5,6]. I3C also affects auxin transporters PIN1 and PIN2 [5] and induces specific autophagy [7].

Using a genetic approach, we previously showed that over-expression of the 30S subunit of the ribosome leads to tolerance to external I3C in Arabidopsis [8]; however, the molecular pathway governing I3C signaling is not fully understood. Here, we utilized an artificial microRNA (amiRNA) library system that targets kinase gene families. This unique approach not only allows for carrying targeted genetics using 1600 amiRNA plasmids targeting different protein kinase families but is also designed to overcome genetic redundancy in large scales [9,10,11]. We report the isolation and analysis of an I3C-tolerant and an I3C-susceptible mutant through screening of a genome-scale amiRNA library. We identified two lines of Arabidopsis with altered responses to I3C: *ICT2*, which showed an increased tolerance, and *ICS1*, which showed hypersensitivity. Molecular analysis revealed that *ICS1* carries an amiRNA targeting three Wall-Associated Kinases (WAK1–3), while *ICT2* carries an amiRNA targeting two phosphatidyniositol-4-phosphate 5 kinases (PIP5k). Physiological, metabolomic, and transcriptomic analyses reveal that these kinases mediate numerous developmental processes and are involved in abiotic and biotic stress responses. These results indicated that WAKs inhibit responses to fungi, while PIP5Ks inhibit responses to bacteria.

## 2. Materials and Methods

The Arabidopsis lines used in this work were all in the Columbia-0 (Col-0) background. A seed library consisting of Arabidopsis lines carrying amiRNAs targeting kinase families was used for screening I3C-response mutants. The information about each individual amiRNA and its locus and sequence, as well as the targeted genes, is found in the DATA file (PHANTOM) which was provided by [12]. Single T-DNA lines of the targeted genes were provided by the Arabidopsis Biological Resource Center (ABRC). The double mutant *cyp79B2/B3* used in this work was provided by [13]. The *ICS1/cyp79B2/B3* double mutant was obtained by crossing between the single mutants ICS1ami0 and *cyp79B2/B3*.

Growth conditions: Seeds were cultivated in Petri plates using medium containing 0.8% agar, half-strength Murashige and Skoog salts (MS), and 1% sucrose at pH 5.7. The Petri plates were placed in chambers at 22 °C under light/dark conditions of 16 h white light at 75 µmol m^−2^ s^−1^ and 8 h darkness at 55% relative humidity. For root phenotype experiments, plates were placed vertically in the chambers. For I3C assays, 500 μM I3C was added to the MS medium, and plates were kept in the growth chamber under the same conditions as above.

Root analysis: Roots were imaged by scanning using an Epson WorkForce GT-1500 scanner (EPSON, Suwa, Nagano, Japan) at a resolution of 600 dpi. Images obtained were used to measure the root length by means of the ImageJ software v 1.8.0. Throughout these studies, we have noticed great variability in root lengths, apparently due to variations in batches of I3C and MS media. While we did consider showing relative inhibition levels rather than absolute numbers, we chose to show the real data.

Statistics: All experiments were repeated at least three times. The variance between the groups that were statistically compared was similar. The samples were analyzed using the Student’s *t*-test. We considered *p* < 0.05 as significant.

Response to biotic stress: *Botrytis cinerea* spores were diluted to 5 × 10^5^ spores mL^−1^ in 0.53 potato dextrose broth. Droplets (7 µL) of 0.53 potato dextrose broth with 105 *B. cinerea* spores were deposited on leaf surfaces of 4-week-old Arabidopsis plants (three leaves per plant). After incubation of the inoculated plants at high humidity for 4 days, the size of the disease lesion was measured. At least 15 lesion diameters were evaluated for each independent treatment (five plants). The lesion area was then measured with ImageJ.

Five-week-old Arabidopsis plants (Col-O, *ICT2*, and *ICS1*) were inoculated with *Pseudomonas syringae* by infiltration with a suspension (1 × 10^5^ CFU mL^−1^) of Pst in a 10 mM MgCl_2_ solution using a needleless syringe. Three leaf discs (diameter, 1 cm) were sampled at 0, 2, and 4 days post-inoculation (dpi) from five inoculated plants and ground in 1 mL of 10 mM MgCl_2_. Samples were then 10-fold serially diluted and plated on LB plates supplemented with 25 mM rifampicin. The colony counts were recorded 2 days after incubation at 28 °C.

Preparation of indole and benzoxazinoids metabolites: Indole-3-carbinol (I3C), indole-3-acetic acid (IAA), indole-3-acetonitrile (IAN), 3,3′-diindolylmethane (DIM), indole-3-carboxaldehyd (I3CxA), and methylindole-3-carboxylate (Mi3Cx) (Sigma, Jerusalem, Israelwere dissolved in dimethyl sulfoxide (DMSO) to produce 1 M solutions and stored in the dark at −20 °C.

Preparation of chemical solutions: For responses to abiotic stress agents, a solution of 100 mM NaCl was prepared as the salt stress, 10 nM H_2_O_2_ solution as the oxidative stress, and 200 nM Mannitol solution as the drought stress by dissolving in water. All chemicals were added to the MS media after autoclaving.

Biochemical and metabolic analysis: Determination of uptake rates of I3C and endogenous levels of I3C

WT, Cyp79b2/b3, *ICT2*, and *ICS1* lines were exposed for 2 h to either 0.05% DMSO or 400 µM I3C (standard, sigma I7256/Merck) following 2 weeks of growth in liquid MS (long day, 22 °C, humidity 50%), ashed 3 times with fresh DDW, and 4 seedlings were pulled together and frozen in liquid nitrogen and lyophilized. Dry samples were powdered with 2 stainless-steel balls in a beater (1 min, MM 400, Retsch). One mg of each sample was extracted with 80% methanol in 0.2% formic acid (500 µL), containing ethylparaben (1 ng/mL) (standard, sigma PHR1011/ Merck) as internal standard (IS), by shaking (20,000 rpm, 65 °C, 15 min, Thermomixer C, Eppendorf). Then, the mixtures were centrifuged (14,000× *g*, 10 min) and supernatants were collected and placed in LC-MS vials. Standard curve mixtures 0.001–10 µg/mL of I3C and DIM (standard, sigma D9568/Merck) were prepared in 80% methanol/0.2% formic acid, containing 1 ug/mL of ethylparaben as IS. The amount of I3C absorbed from the media was calculated by subtracting internal I3C levels in untreated samples from internal I3C levels in treated samples, done by Alexander Brandis from the Weizmann Institute of Science.

LC–MS/MS analysis was performed on an Acquity UPLC system and triple quadrupole Xevo TQ-S (both Waters). TargetLynx (Waters, Milford, MA, USA) was applied for quantitation based on standard curves.

LC parameters: Acquity BEH C18 column (2.1 × 100 mm, 1.7 µm; Waters) at 35 °C. Gradient with mobile phases A—0.01% aqueous formic acid and B—0.01% formic acid in acetonitrile: 0 min, 20%B; 3 min, 100%B; 4 min, 100%B; 4.5 min, 20%B; 7 min, 20% B. Injection volume 2.0 µL, flow rate 0.3 mL/min. Retention times: 2.25 min for I3C, 2.96 min for DIM, and 2.18 min for IS.

MS/MS parameters: electrospray ionization in positive-ion mode, desolvation temperature, 400 °C; desolvation gas flow, 600 L/h; cone gas flow, 150 L/h; nebulizer pressure, 7 Bar; capillary voltage, 3.56 kV, cone voltage 44 V, with argon 0.10 mg/min as the collision gas. The MRM transitions: 130.1 > 77.0 and 130.1 > 103.1 with collision energy (CE) 22 V in both for I3C and DIM; 167.1 > 95.0 and 167.1 > 139.1, with CE 13 and 10 V, respectively, for IS.

Metabolomic analysis: Dried plant powder ~20 mg was extracted using 0.6 mL Methanol:DDW (80:20, *v*:*v*) containing 3 µg/mL of C13 and N15 labeled amino acids mix (Sigma-Aldrich, St. Louis, MO, USA, 767964/Merck) as internal standards. The tubes were vortexed for a minute, then sonicated for 30 min in an ice-cold sonication bath (taken for a brief vortex every 10 min), vortexed again, and then 200 µL of extract was centrifuged at 13,000 rpm at 4 °C for 10 min. Supernatant was transferred into the vials for analysis.

Metabolic profiling of the semipolar phase was performed using a Waters ACQUITY UPLC system coupled to a Vion IMS QTof mass spectrometer (Waters Corp., Milford, MA, USA). The chromatographic separation was performed on an ACQUITY UPLC BEH C18 column (2.1 × 100 mm, i.d., 1.7 μm) (Waters Corp., Milford, MA, USA). The mobile phase A consisted of 95% water (UPLC grade) and 5% acetonitrile, with 0.1% formic acid; mobile phase B consisted of 100% acetonitrile with 0.1% formic acid. The column was maintained at 35 °C and the flow rate of the mobile phase was 0.3 mL*min^−1^. Mobile phase A was initially run at 100%, and it was gradually reduced to 72% at 22 min, followed by a decrease to 60% at 22.5 min and 0% at 23 min. Then, mobile phase B was run at 100% till 26.5 min, and mobile phase A was set to 100% at 27 min. Finally, the column was equilibrated at 100% A till 28 min. The MS parameters were as follows: the source and desolvation temperatures were maintained at 120 °C and 350 °C, respectively. The capillary voltage was set to 2 kV; cone voltage was set to 40 V. Nitrogen was used as desolvation gas and cone gas at the flow rate of 700 L*h^−1^ and 50 L*h^−1^, respectively. The mass spectrometer was operated in full scan HDMSE negative ionization, over a mass range of 50–2000 Da. For the high energy scan function, a collision energy ramp of 20–80 eV was applied, and for the low energy scan function, 5 eV was applied. Leucine–enkephalin was used as a lock-mass reference standard.

The metabolomic analysis was carried out by the Metabolic Profiling Unit of the Weizmann Institute of Science. Briefly, LC-MS data were analyzed and processed with UNIFI (Version 1.9.4, Waters Corp., Milford, MA, USA). The putative identification of the different semipolar species was performed by comparing the accurate mass comparison, fragmentation pattern, and ion mobility (CCS) values of theoretical structures from an in-house generated library. Data were normalized using internal standards and sample weights.

RNA-Seq: Three lines were used for NGS: Col-0, *ICT2*^ami0^, and *ICS1^ami0^*. The seedlings were grown hydroponically for 10 days. Three biological pooled samples (20 seedlings per sample) were collected for each treatment. In all treatments, solutions of DMSO and 400 µM of I3C were prepared in MS medium and added to the liquid medium of hydroponic growth, which was incubated for 2 h. All samples were collected at the same time of day to eliminate any circadian effect.

Roots were excised and RNA extracted with TRIzol using the Zymo RNA mini prep quick kit (R2072). RNA (1500 ng) was used for library preparation. Twenty-four RNA libraries were generated using the Illumina TruSeq RNA Library Preparation Kit v2 according to the manufacturer’s protocol and sequenced on an Illumina HiSeq2500, with 50 single-end runs. An average of 20 million reads were sequenced per sample. Further analyses were carried out as described previously [8]. 

Confocal microscopy

Five-day-old seedlings were transferred to liquid MS containing 500 µM I3C for 2 h. After treatment, seedlings were submerged in 0.005 mg mL^−1^ propidium iodide in double-distilled water, placed on microscope slides, and imaged using a Zeiss LSM780 confocal microscope with 920/NA 0.8. The fluorescence emissions were collected between 590 and 720 nm (band pass) for propidium iodide, and between 520 and 580 nm (band pass) for green fluorescent protein (GFP), and between 593 to 650 nm for red fluorescent protein (RFP). Fluorescence was quantified using ZEN 3.5 blue edition software (https://www.micro-shop.zeiss.com/).

Amplification of amiRNA fragments: Young leaves of *A. thaliana* plants were ground in liquid nitrogen and genomic DNA was extracted using the CTAB method (Murray and Thompson, 1980). PCR was performed to amplify the amiRNA fragments in *ICT2^ami0^* and *ICS1^ami0^* using a pair of specific primers:

amiRNA-F: 5′CGTAAGGGATGACGCACAATC 3′

amiRNA-R:5′ATGCGATCATAGGCGTCTCG3′.

The PCR conditions were 10 min at 95 °C, and then 30 cycles of 94 °C for 30 s, 55 °C for 30 s, and 72 °C for 180 s, and then ending after 10 min at 72 °C to complete the elongation.

(caaacacacgctcggacgcatattacacatgttcatacacttaatactcgctgttttgaattgatgttttaggaatatatatgtagagagagcttccttgagtccattcacaggtcgtgatatgattcaattagcttccgactcattcatccaaataccgagtcgccaaaattcaaactagactcgttaaatgaatgaatgatgcggtagacaaattggatcattgattctctttgattggactgaagggagctccctctctcttttgtattccaattttcttgattaatctttcctgcacaaaaacatgcttgatccactaagtgacatatatgctgccttcgtatatatagttctggtaaaattaacattttgggtttatctttatttaaggcatcgccatg).

The mismatch zones represent the sequence of the 22 nt replaced in the original MIR319a miRNA. The sequence then was looked up in the PHATOM file provided by [12].The sequence found to be matched in this data file leads to the target sequences and locus of the genes found to be silenced by base-pairing with the amiRNA sequence. Then, the gene locus name was used to search in the TAIR website (https://www.arabidopsis.org/).

RNA Production: RNA was purified from whole seedlings. Total RNA was isolated by TRI reagent (Ambion) according to the Trizol RNA Isolation Protocol.

cDNA extraction: The cDNA was synthesized using a cDNA Synthesis Kit (Quanta bio) according to the manufacturer’s instructions.

RT-PCR conditions: The Quantabio Kit (95047-100) was used to synthesize the cDNA from the total RNA. Each RT-qPCR reaction was set up in a 15 μL volume containing 0.6 μL of cDNA, 0.4 μL of gene-specific primers, 7.5 μL of SYBR (Quantabio 65072-012), and 6.1 μL of sterile distilled water. Actin and PPR (AT1G49240 and AT1G62930) were used as the reference genes. Each reaction included three biological replicates, which were analyzed via StepOne software v2.3, using the 2^−ΔΔCt^ method for relative abundance calculation. The experiment was repeated three times.

Cloning: The amiRNA inserts were designed using the WMD3 web microRNA designer. The inserts were cloned in the sense orientation into the binary vector pMA-RQ (AmpR). LR cloning using the gateway protocol was performed to transfer the amiRNA fragments from the entry plasmid to the destination plasmid pGW402. The final constructs were electroporated into Agrobacterium tumefaciens. Col-0 plants were used for transformation with Agrobacterium. The WAK1–3 and PIP5K7,9 coding sequences were amplified and cloned in the sense orientation into the binary vector pGW405:GFP, pGW454:RFP, and pGW545:CFP between the 35S-Ω promoter containing the translation enhancer signal and the Nos terminator, generating the constructs pGW405:WAK1-GFP, pGW454:WAK2-RFP, pGW545:WAK3-CPP, pGW454:PIP5K7-RFP, and pGW405:PIP5K9-GFP.

T-DNA lines were obtained from the SALK and SAIL libraries [14]: WAK 1- AT1G21250: SALK_107175.30.60.x, WAK2- AT1G21270: SAIL_12_D05 (CS870117), WAK 3- AT1G21240: SALK_071999.55.50.x, PIP5K7- AT1G10900: SALK107796, and PIP5K9- AT3G09920: Salk_047851.

## 3. Results

Isolation and phenotypic characterization of I3C-response mutants

An Arabidopsis transgenic amiRNA library was screened for I3C-tolerant and -susceptible mutants. The library contains 1600 lines expressing various amiRNAs targeting different protein kinase families [12] and allows to carry target 2–10 kinase genes from the same family, thus, overcoming potential genetic redundancy. Arabidopsis thaliana ecotype Col-0 was used as the wild-type (WT) control as it was the background line of the library. About 550 lines were screened in this work using a 500 µM I3C root elongation assay [8]. Candidate lines were identified as having visibly longer or shorter roots compared to the WT.

**Figure 1:**
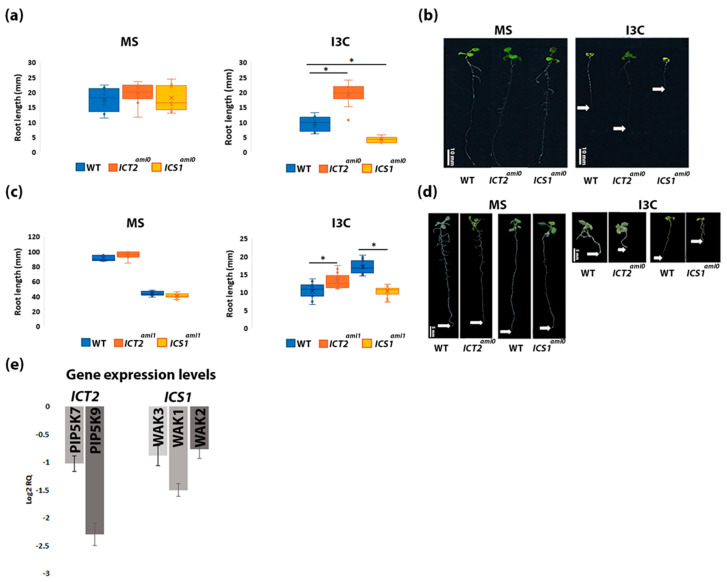
*ICT2* and *ICS1* are tolerant or sensitive to I3C in root growth assays. The data show the average root lengths of two sets of 30 seedlings: (**a**) *ICT2^ami0^* and *ICS1^ami0^*, and (**c**) *ICT2^ami1^* and *ICS1^ami1^* lines. These seedlings were germinated on MS with and without 500 µM I3C added. Corresponding representative seedlings are displayed in panels (**b**,**d**) for both the original and generated lines, respectively, after 14 days of growth on the aforementioned media. The scale used for panel (**b**) is 10 mm, while panel (**d**) is scaled at 5 mm. White arrows indicate root tips. The asterisk denotes a statistically significant difference between the groups, as determined by a *t*-test (*p* < 0.05). (**e**) Gene expression levels of *ICT2* and *ICS1* amiRNA lines. *ICT2* targets PIP5K7 and PIP5K9 while *ICS1* targets WAK1, WAK2, and WAK3. Values are presented in log2-fold relative to WT levels. Actin was used as a standard, *n* = 3.

We identified two amiRNA lines, *indole-3-carbinol tolerant 2 (ICT2)* and *indole-3-carbinol sensitive 1 (ICS1)*, with altered root elongation in response to exogenous indole-3-carbinol (I3C) treatment. *ICT2* is tolerant to I3C, while *ICS1* is susceptible to I3C (Figure 1a,b).

The amiRNA transgene was isolated from each line and sequenced. Each line expresses a unique amiRNA, with *ICT2^ami0^* expressing an amiRNA sequence that targets genes encoding two members of the phosphatidylinositol 4-phosphate 5-kinase family (PIP5K7 and PIP5K9), and *ICS1^ami0^* expressing an amiRNA sequence that targets genes encoding three members of the Wall-Associated Kinase family (WAK1, WAK2 and WAK3).

To verify the phenotypes of *ICT2^ami0^* and *ICS1^ami0^*, additional amiRNA lines carrying different amiRNA sequences and targeting the same kinases were generated (termed *ICT2^ami1^* and *ICS1^ami1^*). Phenotypic examination of the new transgenic plants confirmed the I3C-sensitive phenotypes of *ICS1^ami0^* are indeed due to the presence of an amiRNA targeting WAK1, WAK2, and WAK3, and confirmed the I3C-tolerant phenotypes of *ICT2^ami0^* are indeed due to the presence of an amiRNA targeting PIP5K7 and PIP5K9 (Figure 1c).

Gene expression levels of the predicted amiRNA targets were measured by RT-PCR. As seen in Figure 1e, steady-state levels of PIP5K7 and PIP5K9 are reduced in *ICT2*, and steady-state levels of WAK1, 2, and 3 are reduced in *ICS1*, confirming that the amiRNAs lead to reduced levels of the predicted targets.

After genotypic and phenotypic validation, the lines *ICT2^ami0^* and *ICS1^ami0^* were used for the rest of the experiments in this research.

The response in *ICT2* is specific to I3C

The results above show that *ICT2* and *ICS1* have altered growth responses to exogenous I3C compared to WT. This could be a result of an alteration in response specifically to I3C, or alternatively, the mutants could have altered responses to other chemicals as well.

**Figure 2:**
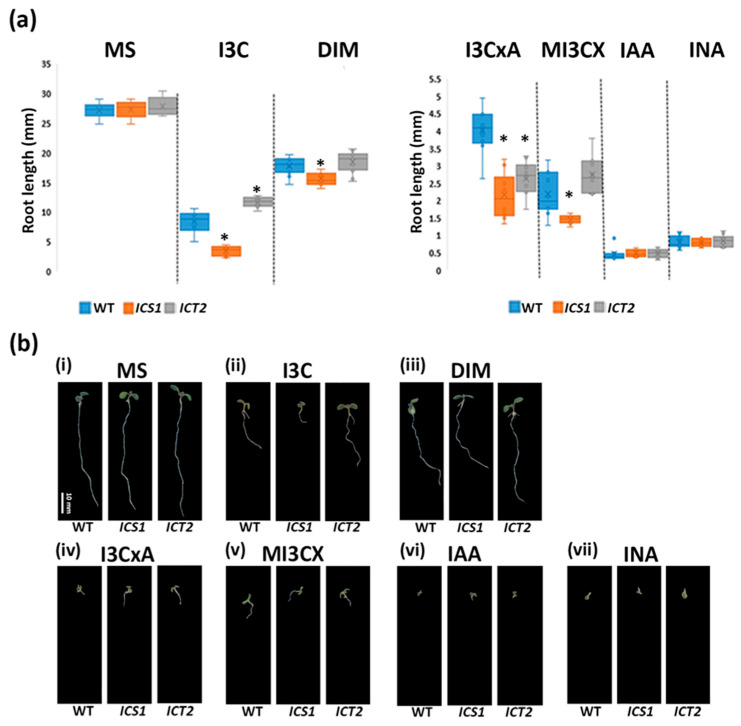
The response in *ICT2* is specific to I3C. (**a**) The graph illustrates the average root lengths of 15 seedlings each of the WT, *ICT2*, and *ICS1* grown on various I3C derivatives. (**b**) Representative 7-day-old seedlings of WT, *ICS1*, and *ICT2* (displayed left to right, respectively) are shown. These seedlings were grown on different media: (**i**) Murashige and Skoog medium (MS), (**ii**) 500 µM I3C, (**iii**) 200 µM Diindolylmethane (DIM), (**iv**) 300 µM I3CxA, (**v**) 375 µM MI3CX, (**vi**) 200 nM indole-3-acetic Acid (IAA), and (**vii**) 160 µM indole-3-Acetonitrile (INA). Scale bar 10 mm. The asterisk denotes a statistically significant difference between the groups, as determined by a *t*-test (*p* < 0.05). Interestingly, I3C appears to mitigate IAA-induced inhibition in both the *ICT2* and *ICS1* lines.

*ICT2* and *ICS1* were monitored for growth responses to different I3C derivatives. For this purpose, we germinated *ICT2*, *ICS1*, and WT seedlings on MS media containing 200 nM IAA, 160 µM INA, 200 µM DIM, 200 µM I3CxA, and 375 µM MI3CX. MS plates and plates with MS containing 500 µM I3C were used as controls. The roots of the mutants were measured and compared to the WT, and the results are presented in Figure 2. *ICT2* was tolerant only to I3C, while *ICS1* was also sensitive to three I3C breakdown products, DIM, I3CxA, and MI3CX. Interestingly, *ICT2* was hypersensitive to I3CxA. Thus, while the I3C tolerance phenotype in *ICT2* appears specific, *ICS1* is hypersensitive to other glucosinolate derivatives.

I3C rescues IAA inhibition in *ICT2* and *ICS1*

I3C and indole-3-acetic acid (IAA) have similar structures, with the difference being that I3C has a terminal hydroxyl group whereas IAA has a terminal carboxylic acid. IAA interacts with the auxin-binding site of the TIR1 receptor [15,16], thus perturbing the interaction of TIR1/auxin-signaling F-Box (AFBs) with auxin/3-indoleacetic acid (Aux/IAAs) proteins [17]. I3C negatively affects auxin signaling by competing with IAA for binding to TIR1 [5,6].

Inhibition of root growth is a typical phenotype of plants exposed to exogenous auxin [18]. In *A. thaliana* WT seedlings, I3C inhibits the inhibitory effect of exogenous IAA on root elongation, thus causing a partial rescue of IAA-mediated root length inhibition [5]. Based on this, we explored if the changes in I3C sensitivity in *ICT2* and *ICS1* also influence the ability of I3C to antagonize IAA signaling.

**Figure 3:**
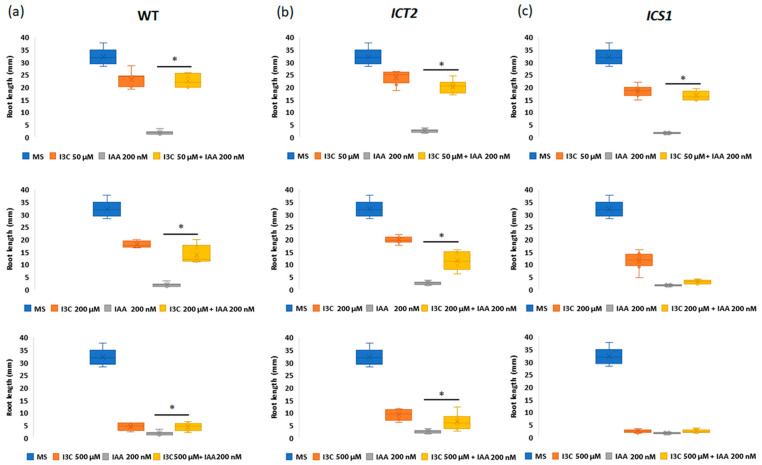
Both *ICT2* and *ICS1* retain I3C inhibition of IAA signaling. WT (**a**), *ICT2* (**b**), and *ICS1* (**c**), were grown on mixtures of three concentrations of I3C (50—top row, 200—middle row, and 500 µM—bottom row) and with or without 200 nM IAA. *n* = 30. The asterisk denotes a statistically significant difference between the groups, as determined by a *t*-test (*p* < 0.05).

**Figure 4:**
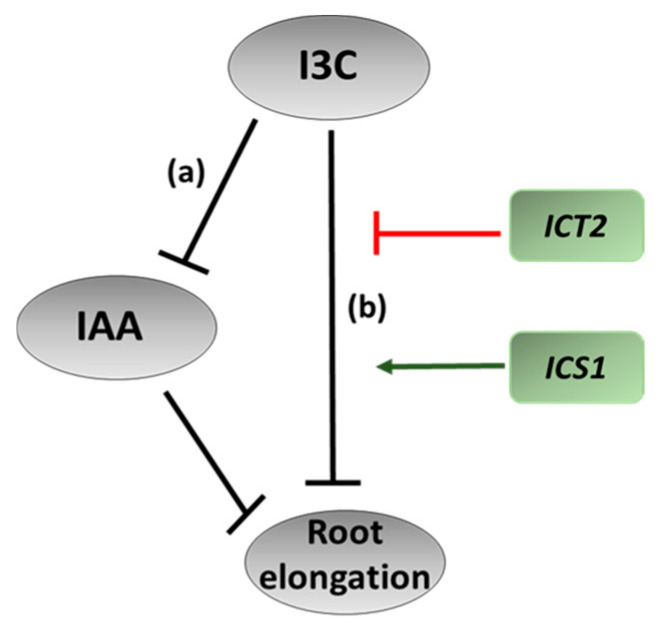
Proposed model for the effect of I3C on root elongation. I3C influences root elongations through two independent pathways. The first (**a**) is through IAA, where I3C inhibits the inhibitory effect of IAA on root elongations. In the second (**b**), I3C directly inhibits root elongation in an IAA-independent manner. *ICS1* potentiates this pathway (green arrow), while *ICT2* inhibits (red line) it. The concentration of I3C needed for the IAA-dependent pathway (**a**) is much less than the direct inhibitory pathway (**b**).

For this purpose, we monitored the root lengths of seedlings grown for 14 days on a medium containing various concentrations of auxin, I3C, or both. As expected, in all lines, roots grown in the presence of auxin were significantly shorter than the roots of plants grown on MS (Figure 3). However, WT seedlings, or either mutant, grown in the presence of both 50 µM I3C and auxin exhibited roots that were longer than those grown solely on auxin, indicating that in the mutants, the ability of I3C to antagonize the effect of exogenous auxin is unimpaired. However, differences in sensitivity were seen. While for WT and *ICT2*, I3C partially rescued IAA-induced root inhibition in all concentrations tested (50, 200, and 500 µM), *ICS1* rescue of IAA-induced root inhibition was obtained only for the lowest concentration (50 µM) I3C (Figure 3). At higher concentrations, I3C itself led to root-growth inhibition in this hyper-sensitive mutant. Thus, similar to *ICT1* [8], the mechanism of action of the PIP5K and WAK kinases on I3C signaling is not through the I3C-IAA Tir1-mediated pathway (illustrated in Figure 4). In this parallel pathway, *ICT2* is inhibitory, resulting in a long root phenotype, and *ICS1* is excitatory, resulting in a short root phenotype.

*ICS1* has elevated levels of internal I3C

**Figure 5:**
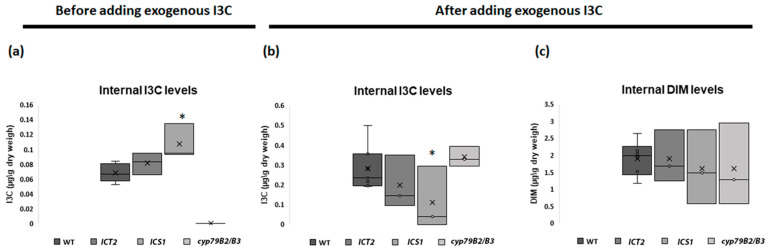
The I3C-sensitive *ICS1* line contains elevated levels of endogenous I3C. Shown are the endogenous levels and uptake rates of I3C in *ICT2* and *ICS1*. (**a**) A box plot shows the endogenous levels of I3C in *ICT2* and *ICS1* and the control line *cyp79B2/B3*. (**b**) The box plot illustrates the amount of internal I3C following exposure to exogenous I3C in the growth medium. The amount of I3C absorbed from media was 0.21 µg\g in WT, 0.11 µg\g in *ICT2*, 0.003 µg\g in *ICS1*, and 0.39 µg\g in *cyp79B2/B3*. (**c**) The box plot represents the levels of Diindolylmethane (DIM), a breakdown product of I3C, for the indicated genotypes. Each box graph is annotated with an asterisk where the differences between the lines and WT are statistically significant, as determined by a *t*-test (*p* < 0.05).

We hypothesized that the IAA-independent mechanism could result either from changes in levels of endogenous I3C, or in uptake of the exogenous I3C. To test these hypotheses, we measured both internal endogenous I3C levels and the amount of I3C absorbed from the media. The *cyp79B2/3* double mutant lacking the two enzymes mediating the synthesis of tryptophan-derived indole GLs [19] was used as a control (Figure 5).

As expected, no I3C was produced in the double mutant *cyp79B2/3*, which is defective in indole glucosinolates synthesis, though it still maintained WT uptake levels of exogenous I3C (Figure 5a,b). The I3C-sensitive *ICS1* line contained elevated levels of endogenous I3C, which could explain the sensitivity to exogenous I3C. However, this line also exhibited a reduced uptake of exogenous I3C (Figure 5b). No changes in I3C levels or uptake were detected for *ICT2*. No changes were detected in the levels of the I3C-breakdown product 3,3′-diindolylmethane (DIM) (Figure 5c).

**Figure 6:**
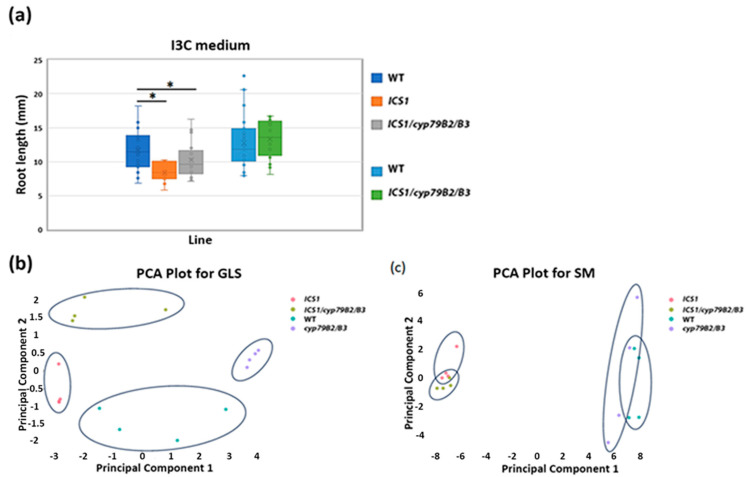
Susceptibility to I3C is rescued in the double mutant *ICS1/cyp79B2/B3*. (**a**) The graph shows the root length for the indicated genotypes. The whisker graph compares the root lengths of *ICS1* and *cyp79B2/B3* single mutants with those of the triple mutant *ICS1/cyp79B2/B3*. All plants were grown on MS medium supplemented with 500 µM I3C. Asterisks denote significant differences in root length, as determined by a *t*-test (*p* < 0.05). A principal component analysis of (**b**) GL profiles and (**c**) SM profiles presenting different patterns between the four tested lines and partial overlap respectively.

The results above suggest that the tolerance in *ICT2* is not connected to reduced uptake or reduced I3C levels. On the other hand, I3C sensitivity in *ICS1* could be a result of increased levels of internal I3C. To test this hypothesis, we crossed *ICS1* with *cyp79B2/B3*, which lacks endogenous I3C. T_3_ seedlings were germinated on I3C and root lengths were measured. The results in Figure 6 show that *ICS1/cyp79B2/B3* has WT levels of I3C sensitivity, indicating that the susceptibility of *ICS1* to I3C is rescued by reducing the amount of internal I3C.

The lines above were further analyzed for glucosinolate (GL) content to determine whether the profile of GLs overall is altered in the triple mutant *ICS1/cyp79B2/B3*. The results in Figure 6b present a principal component analysis graph of the GL profiles in the tested lines. The four lines are clearly differentiated from each other in this analysis. This is distinct from the results of non-GL secondary metabolite (SM) profiles, where *ICS1* overlaps with the triple mutant *ICS1/cyp79B2/B3*, forming one group, while the double mutant *cyp79B2/B3* together with the WT form another group (Figure 6c). Taken together, these results indicate that *ICS1* strongly influences both the GL and SM profiles. As the GL content is, as expected, strongly influenced in *cyp79B2/B3*, the GL profiles of the triple mutant separate into a unique grouping between the two parental lines *ICS1* and *cyp79B2/B3*. For other SM, the triple mutant behaves as *ICS1*. Therefore, both the GL and SM profiles are influenced by the downregulation of WAKs in the mutant *ICS1*.

Glucosinolates and other secondary metabolite profiles in *ICT2* and *ICS1*

**Figure 7:**
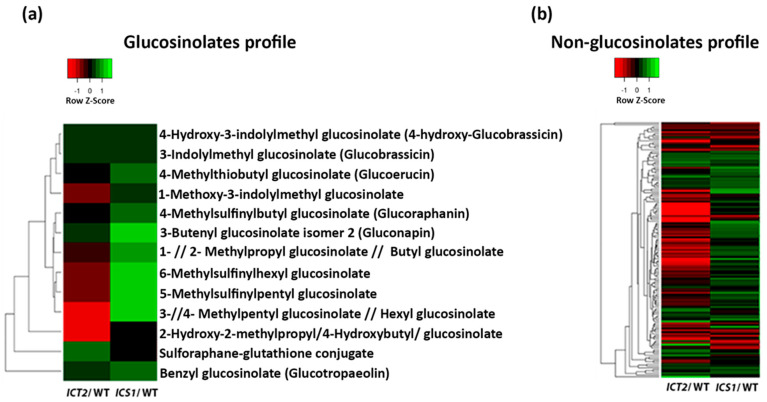
Metabolites content in *ICT2* and *ICS1*. This figure visualizes the profiles of secondary metabolites in *ICT2* and *ICS1* using heat maps. The color intensity in the heat maps represents the concentration of each metabolite, with values presented in log2 scale. (**a**) This heat map displays the profile of glucosinolates (GLs) in *ICT2* and *ICS1* mutants compared to the WT. (**b**) The second heat map presents the profile of non-glucosinolate (non-GL) secondary metabolites in the same mutants relative to the WT. Data are taken from Supplementary Table S1.

Since endogenous I3C levels were altered in *ICS1*, we checked whether the profile of glucosinolates overall was also altered in this line. The results in Figure 7a show that *ICS1* had a higher GL profile relative to the WT, including increases in aromatic, aliphatic, and indolic glucosinolates. On the other hand, the levels of most GLs in *ICT2* are lower. Together with this, five aliphatic and aromatic GLs are significantly different between *ICT2* and the WT, three with higher relative abundance in the mutant, and the other two with lower relative abundance.

We also analyzed the profile of non-GL metabolites in these lines. The results present different patterns between the lines. Relative to WT, *ICT2* contains lower levels of numerous non-GL secondary metabolites compared to ICS1 (Figure 7b).

Changes in gene regulation following I3C treatment

We previously showed that I3C treatment influences the expression of several thousand genes in WT Arabidopsis [8]. Transcriptome analysis was performed to gain an understanding of the intersection with I3C signaling and the pathways influenced by WAK1–3 and PIP5K7/9. Transcriptome profiles were generated for WT, *ICS1*, and *ICT2*, before and following exposure to I3C. Following initial data analysis, several thousand genes were again identified as I3C-responsive in WT, as reflected by the log2 (fold change) values, representing groups of genes with similar ontologies (e.g., hormonal responses, stress responses, etc.), as reported earlier.

**Figure 8:**
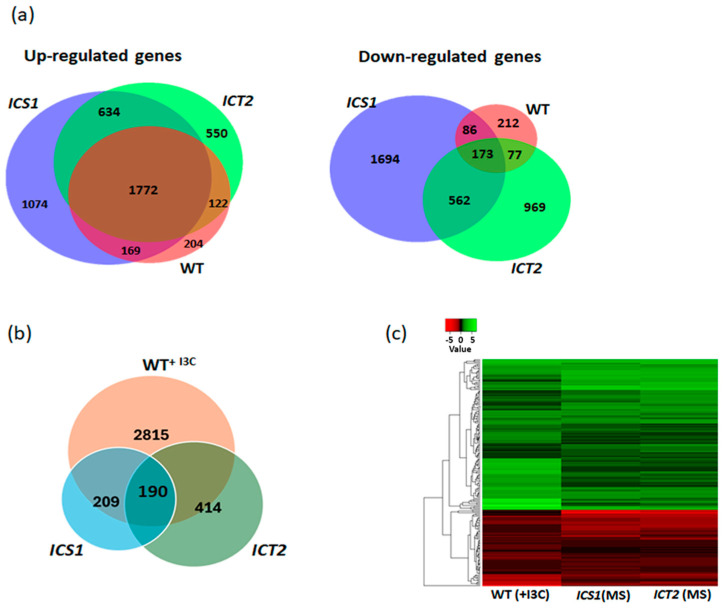
Transcriptomic analysis of the influence of I3C on gene expression in WT and I3C mutant lines. This figure provides insights into the impact of I3C on gene expression in wild type (WT) and I3C mutant lines *ICT2* and *ICS1*. (**a**) A Venn diagram illustrates the overlap between genes upregulated and downregulated in the WT, *ICT2*, and *ICS1* lines (presented in different colors) following I3C treatment. Notably, 1772 genes are commonly upregulated and 173 genes are commonly downregulated across the different lines. (**b**) A Venn diagram illustrates the overlap between misregulated genes in treated WT and untreated *ICT2* and *ICS1*, with a common 190 primed genes. (**c**) A heatmap displays the genes that are primed in the WT line treated with I3C and in the untreated *ICT2* and *ICS1* mutant lines.

We checked if the tolerance or sensitivity to I3C is manifested at the transcriptome level. As seen in Table 1, more genes were identified as I3C-responsive in *ICS1* than in *ICT2*, consistent with the hypersensitivity to I3C in *ICS1*. While there was a large overlap in the upregulated genes in the three lines (1772 common genes, representing 49–78% of the upregulated genes, depending on the line), very few genes (173, representing 7–35% of the downregulated genes, depending on the line) were commonly downregulated (Figure 8a). The common upregulated genes included genes involved in indole GL metabolism, toxin catabolic process pathways, response to hormones and to abiotic stresses, defense responses to bacteria and fungi, and programmed cell death. Downregulated genes were mainly involved in abiotic and defense responses.

In the absence of I3C, *ICT2* and *ICS1* exhibited the misregulation of a number of genes whose expression levels are influenced by I3C in the WT. Thus, these genes, the majority of which are involved in responses to biotic and abiotic stresses, are referred to as ‘primed’ (Figure 8b). The 322 upregulated primed genes in *ICT2* included genes involved in responses to chitin, auxin, bacteria and fungi, toxin catabolic processes, and phenylpropanoid biosynthesis. The 92 downregulated primed genes included genes involved in lipid localization, regulation of Rab protein signal transduction, phosphorylation of the transmembrane receptor protein tyrosine kinase signaling pathway, and responses to bacteria.

*ICS1* presented 126 upregulated primed genes. These genes are involved in metabolism of carbohydrates, sugar, organic acids, amino acids and carboxylic acids, and in response to abiotic stresses such as radiation, light, temperature, oxidative stress, osmotic stress, and wounding. The 83 downregulated primed genes in *ICS1* are involved in hormone responses (ethylene, abscisic acid, auxin, and jasmonic acid), metabolism of glucose, carbohydrates, amino acids, carboxylic acid and auxin, responses to abiotic stresses such as hydrogen peroxide, light, oxidative stress, temperature and wounding, and responses to bacteria and fungi. Table 2 summarizes the pathways these genes are involved in, and Figure 8c presents a heatmap of the 190 common primed genes in *ICT2* and *ICS1*.

Transcriptome analysis of *ICT2* and *ICS1* in the absence of I3C further revealed that, in addition to the primed genes, numerous other genes are misregulated. As seen in Table 2, gene ontology analysis identified many genes involved in abiotic stress responses and metabolic processes. Thus, WAK1–3 and PIP5K7 and PIP5K9 are clearly involved in mediating numerous developmental, stress, and metabolic processes. Particularly, genes involved in responses to biotic and abiotic stresses are misregulated in both *ICT2* and *ICS1*, suggesting a role of the kinases in mediating the responses to different stresses in the plant.

WAKs and PIP5Ks are involved in mediating abiotic stresses

**Figure 9:**
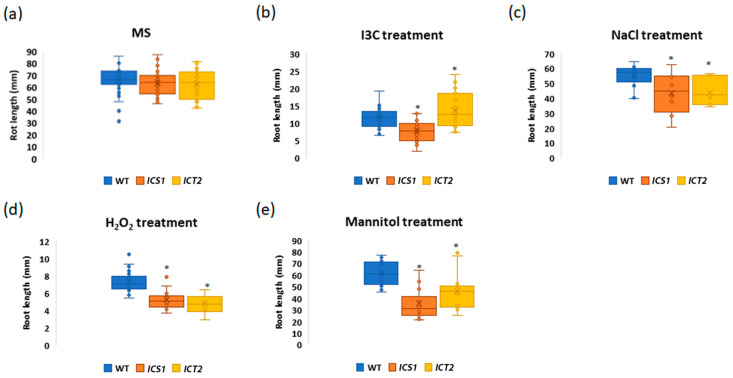
WAKs and PIP5Ks mediate abiotic stress response in Arabidopsis. This figure presents root lengths for WT, *ICT2*, and *ICS1* when exposed to different conditions. Seedlings were germinated on (**a**) standard MS medium or MS supplemented with (**b**) 500 µM I3C, (**c**) 50 mM NaCl (**d**) 1 nM H_2_O_2_, or (**e**) 200 mM Mannitol. An asterisk denotes significant differences in root length, as determined by a *t*-test (*p* < 0.05), *n* = 30.

To assess the correlation between the transcriptome data and physiological phenotypes, we monitored the growth responses in the mutants following exposure to different chemicals to determine whether they were altered in their ability to respond to abiotic stresses. Seeds were germinated on MS media and exposed to 50 mM NaCl (salt stress), 10 mM H_2_O_2_ (oxidative stress), or 200 nM mannitol (drought stress), and analyzed with the root-length assay as above. The results presented in Figure 9 show that both *ICS1* and *ICT2* were more sensitive to NaCl, mannitol, and H_2_O_2_ compared to the WT, indicating that WAK1–3 and PIP5K7 and PIP5K9 are necessary for mediating abiotic stress responses in the plant. Apparently, the downregulation of abiotic stress-response genes in these mutant compromises their response to stress.

*ICT2* and *ICS1* are tolerant to *Pseudomonas syringae* but susceptible to *Botrytis cinerea*

**Figure 10:**
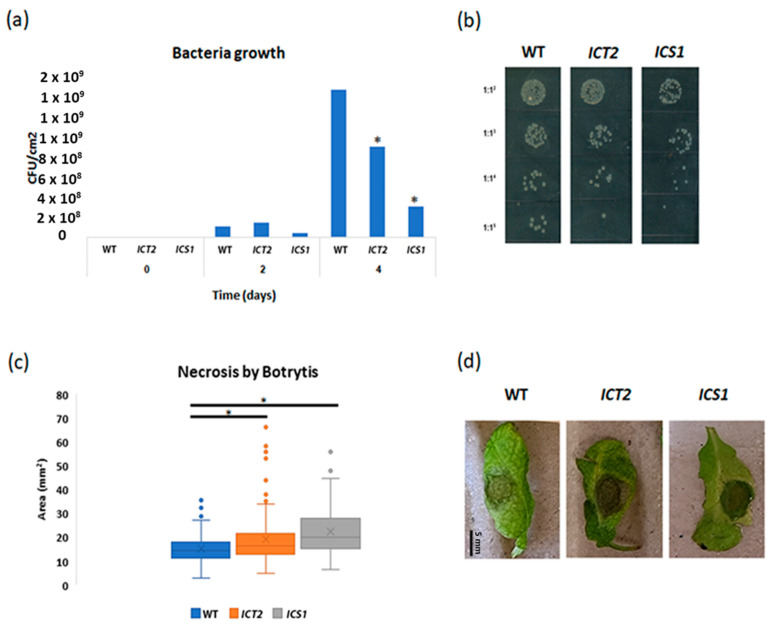
*ICT2* and *ICS1* are tolerant to *Pseudomonas syringae* infection and susceptible to *Botrytis cinerea*. This figure showcases the relative disease resistance and susceptibility of the *ICT2* and *ICS1* lines to specific pathogens. (**a**) A bar graph depicts the number of colonies CFU/cm^2^ in WT, *ICT2*, and *ICS1* plants infected with *Pseudomonas syringae*, measured at two and four days post-infection. The asterisk denotes statistically significant differences as determined by a *t*-test (*p* < 0.05). (**b**) Images represent the growth of *Pseudomonas syringae* colonies in WT, *ICT2*, and *ICS1* plants infected with *Pseudomonas syringae* at different dilutions. (**c**) A whisker chart shows the areas of necrosis measured in leaves from WT, *ICT2*, and *ICS1* four days after infection with *Botrytis cinerea*. * Statistical *t*-test, *p* < 0.05. (**d**) Photographic representation of WT, *ICT2*, and *ICS1* leaves infected with Botrytis.

As the transcriptome analysis identified biotic stress genes as constitutively misregulated in both *ICT2* and *ICS1*, we monitored the response of these lines to pathogen challenge. We infected leaves with *Pseudomonas syringae* and examined the growth of the bacteria. Three to five leaves of each plant were infiltrated with a bacteria solution grown to OD_600_ = 0.002. Three discs from the leaves of different plants for each line were collected and ground in MgCl_2_ at four time points. Five dilutions were prepared from each bacteria solution and sown on LB/antibiotic plates. The plates were kept in 37 °C for 2 days and scanned for colony count. The results shown in Figure 10a indicate that both lines are tolerant to *Pseudomonas syringae* infection.

We further tested the response of these lines to the fungal pathogen *Botrytis cinerea*. Here, leaves were cut from *ICT2*, *ICS1*, and WT and placed in a hermetically sealed box. A 7 µL drop of 105 Botrytis solution mixed with tween-20 was dropped on each leaf. The leaves were scanned after four days and necrosis areas on the leaf were measured. The results presented in Figure 10c show that both lines are hyper-susceptible to Botrytis.

Characterization of WAK1–3 and PIP5K7,9 single mutants and subcellular localization

**Figure 11:**
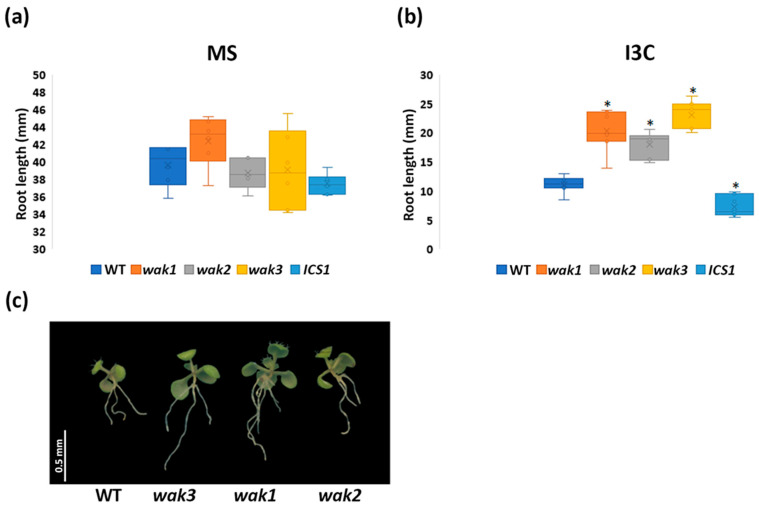
Analysis of T-DNA mutants *wak1*, *wak2*, and *wak3*. Root lengths of homozygous T-DNA lines *wak1*, *wak2*, *wak3*, and triple knock-down line *ICS1^ami0^* grown on (**a**) MS medium and on (**b**) MS medium with 500 µM I3C. * Statistical *t*-test, *p* <0.05. (**c**) Representative 14-day-old seedlings grown on MS medium with 500 µM I3C, scale 0.5 mm.

To determine which, if any, of the WAK genes individually contribute to the I3C-sensitive phenotype of *ICS1^ami0^*, we characterized the response to exogenous I3C in T-DNA lines with insertions in WAK1, WAK2, and WAK3. Homozygous lines for T-DNA insertions were obtained in *wak1*, *wak2*, and *wak3* and qPCR was employed to confirm lack of expression of the specific gene. Surprisingly, T-DNA lines for each of the three genes silenced in *ICS1^ami0^* (WAK1, WAK2, and WAK3) were tolerant to I3C, as exhibited by longer roots than the wild-type when grown on 500 µM I3C (Figure 11). This suggests that the susceptibility to I3C in *ICS1^ami0^* is obtained following the knockdown of the three members of the WAK family; WAK1, WAK2, and WAK3.

**Figure 12:**
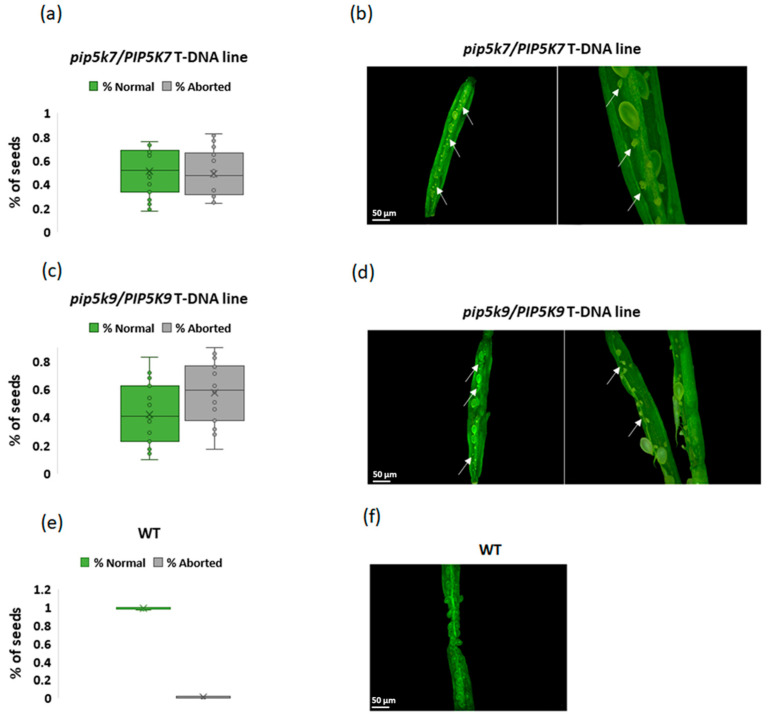
Seed abortion rate in *pip5k7* and *pip5k9*. Percentage of normal and aborted seeds in mature siliques of (**a**) *pip5k7/PIP5K7* and (**c**) *pip5k9/PIP5K9* T-DNA lines and (**e**) WT. Representative mature open siliques of (**b**) *pip5k7/PIP5K7*, (**d**) *pip5k9/PIP5K9*, and (**f**) WT. White arrows indicate undeveloped seeds. Scale bar 50 µm.

To determine which, if any, of the PIP5K7 or PIP5K9 genes individually contribute to the I3Ctolerant phenotype of *ICT2^ami0^*, we attempted to characterize the response to -exogenous I3C in T-DNA lines with insertions in PIP5K7 or PIP5K9. However, no lines homozygous for the T-DNA insertion were obtained for either PIP5K7 or PIP5K9, which raised the possibility that homozygous lines for insertions in these genes could be lethal. To test this possibility, mature siliques from two independent T-DNA lines of both *pip5k7/+* and *pip5k9/+* heterozygous plants were examined and revealed seed abortion with a rate of ~50% (Figure 12). This indicates that homozygous embryos carrying two mutated copies of the genes PIP5k7 and PIP5K9 fail to develop. Thus, both PIP5k7 and PIP5K9 are essential genes.

**Figure 13:**
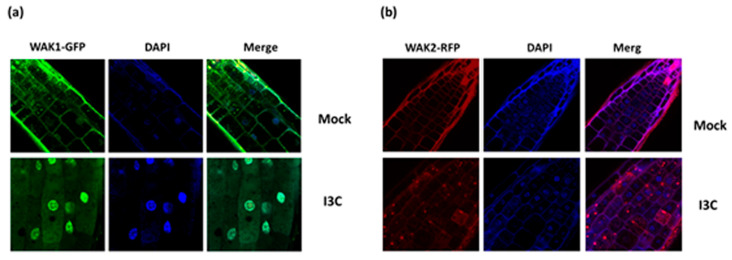
I3C treatment affects the subcellular localization of WAK1 and WAK2. Subcellular localization of (**a**) WAK1 fused to the fluorescent marker GFP and (**b**) WAK2 fused to the fluorescent marker RFP in normal conditions (upper panel) and following I3C treatment (lower panel). The dye DAPI was used to stain the nuclei (blue).

To further characterize the WAK1 and 2 in the I3C response, we attempted to reveal the cellular location of these protein kinases by employing a fluorescence tagging approach. The coding sequence of each gene from a genomic WT cDNA were amplified and cloned in different fluorescent vectors, pGWB405-GFP, pGWB454-RFP, and pGWB545-eCFP, under the control of 35S promoter. Col-0 plants were transformed and T_1_ seeds were germinated on MS plates containing antibiotics for transgenic seedling selection. Seedlings were grown for 5 days with or without treatment with 500 µM I3C for 2 h prior to visualization. The seedlings were imaged using a confocal microscope and the fluorescent dye DAPI was used to stain the nucleus. Figure 13 presents the results of the fluorescent lines WAK1:GFP and WAK2:RFP. As expected, WAK1 and WAK2 were detected in the cell membrane in normal conditions. However, WAK1 was detected in the nucleus following I3C treatment, while WAK2 was seen in small vesicles in the cells. Thus, I3C treatment influences the subcellular localization of WAK1 and WAK2, though the physiological implication of this is unknown.

## 4. Discussion

Previous studies showed that I3C antagonizes the auxin-signaling pathway in Arabidopsis by directly competing with IAA on TIR1 [5,6,7]. To further understand how I3C influences plant metabolism and growth, we applied a genetic approach to isolate mutants with altered responses to I3C. Following a screening of an amiRNA library targeting protein kinase families, we isolated two amiRNA knockdown lines: one with stable tolerance to I3C, termed *ICT2*, and one with stable hyper-susceptibility to I3C, termed *ICS1*. *ICT2* carries an amiRNA targeting two phosphatidyniositol-4-phosphate 5 kinases, PIP5k7 (AT1G10900) and PIP5K9 (AT3G09920). *ICS1* carries amiRNA targeting three Wall-Associated Kinases, WAK1 (AT1G21250), WAK2 (AT1G21270), and WAK3 (AT1G21240).

Several lines of evidence indicate that the altered responses to I3C in *ICT2* and IC*S1* are due to the downregulation of PIP5K7/9 and WAK1–3, respectively. First, additional independent amiRNA lines that target these kinases displayed similar phenotypes to exogenous I3C, indicating that the phenotypes detected are indeed due to the on-target amiRNA activity. Second, all lines studied had decreased expression of the *PIP5K7,9-* or *WAK1–3* kinase-encoding genes (Figure 2). Thus, the tolerance and susceptibility to I3C are unlikely to be due to fortuitous integration of the transgene, but instead to arise directly from the downregulation of the kinases. However, considering the non-straightforward phenotypes of the T-DNA mutants in the individual genes knocked down in *ICT2* or IC*S1*, complementation of amiRNA lines by amiRNA-resistant gene variants could be an appropriate strategy to further characterize the underlying mechanisms of their phenotypes.

Wall-Associated Kinases (WAKs) are a group of receptor-like kinases found in plants. Arabidopsis contains five WAKs (WAK1–WAK5) that share a similar domain structure and function. WAKs are localized to the plasma membrane and play essential roles in plant growth, development, and stress responses [20]. These include perceiving mechanical stress and pathogen invasion by binding to pectin fragments released during cell wall damage or pathogen attack [21]. WAKs are also implicated in various stress responses in plants, including bacterial, fungal, and oomycete pathogens [22], and have been associated with abiotic stress tolerance in Arabidopsis, such as responses to drought, salt stress, and reactive oxygen species (ROS) [23]. Indeed, the transcriptional analysis carried out here correlates with these functions.

Phosphatidylinositol 4-phosphate 5-kinases (PIP5Ks) are a group of kinases that catalyze the phosphorylation of phosphatidylinositol 4-phosphate (PI4P) to generate phosphatidylinositol 4,5-bisphosphate (PI(4,5)P2), a critical phospholipid that serves as a precursor for second messengers and plays a vital role in membrane trafficking, cytoskeletal organization, and signal transduction [24]. PIP5Ks are primarily localized to the plasma membrane, where they regulate the levels of PI(4,5)P2, and are associated with stress responses in Arabidopsis, where they participate in plant defense mechanisms against pathogens and abiotic stresses such as drought and salt stress by modulating phosphoinositide-dependent signaling pathways involved in stress perception and response [25,26]. Again, the transcriptional analysis carried out here correlates with these functions.

What could be the mechanisms by which PIP5Ks and WAKs interface with I3C signaling?

The question of phenotypic specificity is always an issue when dealing with lines that have altered responses to a given challenge. The tolerance phenotype of ICT2, similar to that of another I3C-tolerant line, *ICT1* [8], was most specific to IC3. Indeed, *ICT1* was not tolerant to any other of the chemicals tested, whether they were indole derivatives or auxin. Thus, the downregulation of *PIP5k7* and *PIP5K9* influences an I3C signaling pathway in an as yet unidentified mechanism that is independent of TIR1 signaling. This specificity is perhaps surprising as PIP5Ks are associated with other stress responses in Arabidopsis. For example, these plasma membrane-localized kinases participate in plant defense mechanisms against pathogens and abiotic stresses such as drought and salt stress by modulating phosphoinositide-dependent signaling pathways involved in stress perception and response [25,26].

*ICS1* was not only sensitive to I3C but also to three additional I3C breakdown products, DIM, I3CxA, and MI3CX. This could indicate that the downregulation of the Wall-Associated Kinases allowed an increased uptake of I3C and its derivatives. However, *ICS1* actually had decreased I3C uptake. This line, though, did exhibit increased endogenous I3C levels, and it could be that the increased sensitivity to the I3C breakdown products is an outcome of the increased levels of GLs and SMs in general in *ICS1*. The mislocaliztion of WAK1 and WAK2 from the plasma membrane following a 2 h treatment with I3C further supports the confluence of I3C signaling and WAK function.

Transcript profiling is a powerful unbiased approach to studying the influence of genetic manipulations on phenotype. We employed transcript profiling to study two distinct questions:

1. How are the I3C-related phenotypes correlated with transcriptome?
2. What are the transcriptional outcomes of the loss of PIP5Ks’ or WAKs’ function on the plant?

We previously showed [8,27] that the transcript levels of several hundred genes change following a two-hour exposure to I3C. Many of the same genes misregulated in WT following a two-hour exposure to I3C are also misregulated in *ICT2* and *ICS1* following exposure to I3C. Interestingly, more genes though were misregulated in *ICS1,* which correlates with its increased sensitivity to I3C.

We noticed that many of these same genes are already misregulated in both *ICT2* and *ICS1* under normal growth conditions. That is, genes that are misregulated in WT following exposure to I3C are misregulated in the mutants under normal conditions. Thus, the loss of function of PIP5K7–9 and WAK1–3 apparently leads to a change in gene expression, such that the plant is primed to transcriptionally respond to I3C. These primed genes include those involved in plant responses to biotic stress.

Both *ICT2* and *ICS1* were sensitive to different abiotic stresses, supporting the transcriptomic analysis and earlier studies showing the WAKs and PIPs play a role in mediating abiotic stresses such as mannitol, salt, and H_2_O_2_ [23,26].

Common mechanisms of tolerance to chemical exposure include detoxification and reduced uptake. The amount of internal I3C was significantly higher in *ICS1* compared to WT, where, on the other hand, the I3C amount absorbed from the growing media was significantly lower. These results suggest that the susceptibility of *ICS1* to external I3C could be a result of increased levels of internal I3C rather than I3C uptake from the medium, and this susceptibility could be rescued by reducing the amount of internal I3C.

WAKs and PIP5Ks are also known to be involved in biotic stress responses in plants, including responses to bacterial, fungal, and oomycete pathogens [22,23,24,25,26,28]. Indeed, and in correlation with the transcriptional change, we showed here that both *ICT2* and *ICS1* had altered responses to pathogens. Both lines were tolerant to *Pseudomonas syringae* challenge. Interestingly, the I3C-sensitive line *ICS1* was more tolerant to *P. syringae* than *ICT2*. This supports the transcriptomic analysis where bacterial response genes such as ribonucleases and lysozymes involved in defense response to gram-positive bacteria, globulin binding proteins involved in antibacterial humoral responses, and protein kinases involved in defense responses were upregulated in *ICS1*. Furthermore, we showed that *ICS1* contained higher levels of glucosinolates and other secondary metabolites. These secondary metabolites act as antimicrobial repellents and enhance the defense response of the plant to pathogens []. Interestingly, this tolerance was not broad for all pathogens, as both lines were hyper-sensitive to the fungal pathogen *Botrytis cinerea*.

Among their diverse biological activities, secondary metabolites are known to act as antimicrobials and repellents [29]. The fact that *ICS1* is more tolerant to bacterial infection than *ICT2*, together with the transcriptome analysis of *ICS1* that revealed upregulation in bacterial response genes, could be explained by the higher amount and diversity of GLs and secondary metabolites that *ICS1* produces. According to a yeast-2-hybrid assays online database (thebiogrid.org), WAK2 and WAK3, which are downregulated in *ICS1,* interact with the transcription factors MYB51 and CYP83A1/B1, respectively. MYB51 and CYP83A1/B1 mediate the synthesis pathway of aromatic GLs from the amino acid phenylalanine. Taken together, we could hypothesize that WAK2 and WAK3 have negative effects on MYB51 and CYP83A1/B1, where a loss of function of these two WAKs leads to normal activity of MYB51 and CYP83A1/B1, resulting in higher GL production.

While our motivation was to identify components of the I3C-signaling pathways, it does not appear that WAK1–3 or PIP5K7 or PIP5K9 are directly regulated by I3C. What then might be the mode of action by which the downregulation of PIP5K7 and PIP5K9 leads to I3C tolerance, while WAK1–3 downregulation leads to I3C susceptibility?

Studies have indicated that WAKs are involved in defense responses in plants [30,31,32]. In cells lacking WAK2, the induction and repression of genes involved in cell wall biogenesis and stress responses are blocked, resulting in a short root phenotype and a reduction in vacuolar invertase, raising the possibility that WAKs indirectly control turgor-driven expansion [33]. Since WAK members are shown to be involved in stress responses and in development, the lack of them could be the reason for the obtained phenotype of seedlings exposed to external I3C as a stressor.

A study of kinases such as MAPKs [34] showed that the critical function of MAPKs in plant development is often masked by gene redundancy. Future detailed studies of the single mutants of PIP5K7, PIP5K9, WAK1, WAK2, and WAK3 are expected to reveal additional information about their roles in plants, both in general and in response to I3C treatment.

## Figures and Tables

**Table 1:** Summary the number of I3C-responsive genes in the different lines.

I3C-Responsive Genes	WT	* ICT2 *	* ICS1 *
Upregulated	2267	3078	3649
Downregulated	548	1781	2515
Total	2815	4859	6164

**Table 2:** GO pathways enriched in *ICT2* and *ICS1* in the absence of I3C treatment. “Primed” refers to genes whose expression levels are similarly up- or downregulated in WT following I3C treatment. The highlights call attention to similar gene groupings.

	* ICT2 *		* ICS1 *	
	Primed	Others	Primed	Others
Upregulated genes	Response to bacteria and fungi, chitin Responses to auxin Cell wall loosening and cell size changes. Toxin catabolic process and phenylpropanoid biosynthesis.	Lipid localization and transport.	Abiotic stress response to radiation, light, temperature, oxidative stress, osmotic stress and wounding. Metabolism of carbohydrates, sugar, organic acids, amino acids and carboxylic acids.	Response to bacteria. Transmembrane receptor protein phosphorylation, and regulation of Rab protein signal transduction and Ras GTPase activity. Lipid localization.
Downregulated genes	Responses to bacteria, lipid localization, regulation of Rab protein signal transduction, and phosphorylation of the transmembrane receptor protein tyrosine kinase signaling pathway.	Abiotic stress responses to metal ions, hormones, radiation, light, temperature, oxidative stress, osmotic stress, and wounding. Metabolism of carbohydrate, sugar, organic acid, amino acids, and carboxylic acids. Protein transport and localization.	Responses to bacteria and fungi. Abiotic stress responses to hydrogen peroxide, light, oxidative stress, temperature, and wounding. Responses to ethylene, abscisic acid, and jasmonic acid. Metabolism of glucose, carbohydrates, amino acids, carboxylic acid, and auxin.	Abiotic stress responses to metal ion, salinity, radiation, and water. Vitamin processes and the PS2-associated light harvesting complex 2 process. Lipid oxidation process, negative regulation of post-embryonic development, response to glucose stimulus, and protein transport.

## Data Availability

The original contributions presented in the study are included in the article/supplementary material, further inquiries can be directed to the corresponding author.

## Supplementary Materials

The following supporting information can be downloaded at: https://www.mdpi.com/article/10.3390/biom14101253/s1, Figure S1: Kinetics of inhibition of WT, *ICT2* and *ICS1* root elongation by I3C. (**a**) graphical presentation of root inhibition in WT, *ICT2* and *ICS1* over multiple concentrations of I3C and six-time points. (**b**) WT, (**c**) *ICT2* and (**d**) *ICS1* seedlings grown on different concentrations of I3C. Scale 10 mm; Table S1: Glucosinolates and non-glucosinolates secondary metabolites content in *ICT2* and *ICS1* relative to WT.
