# Supplementary material for: Investigation of the Roles of Phosphatidylinositol 4-Phosphate 5-Kinases 7,9 and Wall-Associated Kinases 1–3 in Responses to Indole-3-Carbinol and Biotic Stress in Arabidopsis Thaliana
Source: Biomolecules. 2024 Oct 3;14(10):1253. doi: 10.3390/biom14101253 (PMC11506499; doi:10.3390/biom14101253)
Supplement: Supplementary file 1 [file biomolecules-14-01253-s001.zip › biomolecules-3207344-Figure S1.pdf]

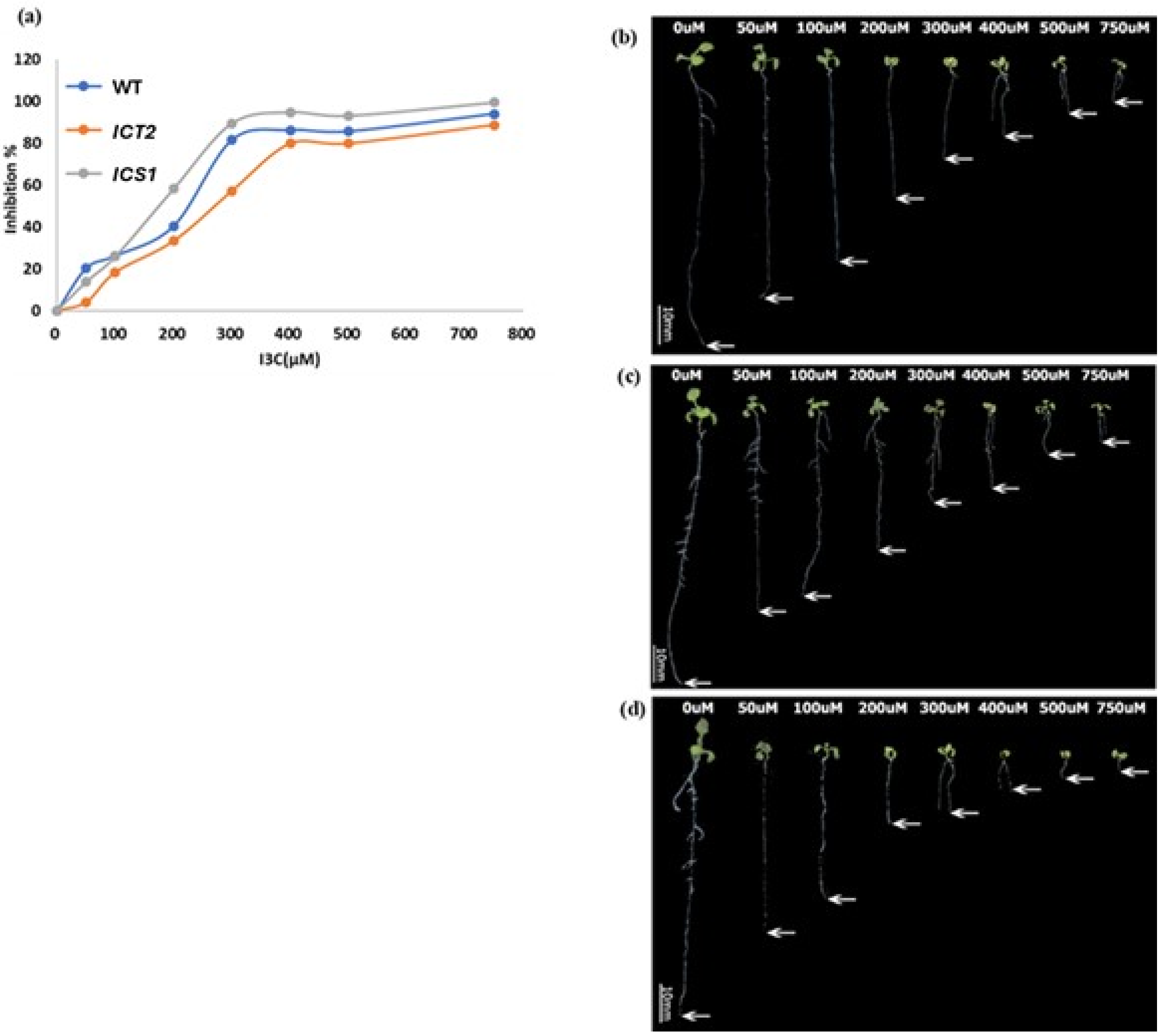

Figure S1. Kinetics inhibition of WT, *ICT2* and *ICS1* root elongation by I3C. (**a**) graphical presentation of root inhibition in WT, *ICT2* and *ICS1* over multiple concentrations of I3C and six-time points. (**b**) WT, (**c**) *ICT2* and (**d**) *ICS1* seedlings grown on different concentrations of I3C. Scale 10 mm.
