# Supplementary material for: Investigation of the Roles of Phosphatidylinositol 4-Phosphate 5-Kinases 7,9 and Wall-Associated Kinases 1–3 in Responses to Indole-3-Carbinol and Biotic Stress in Arabidopsis Thaliana
Source: Biomolecules. 2024 Oct 3;14(10):1253. doi: 10.3390/biom14101253 (PMC11506499; doi:10.3390/biom14101253)
Supplement: Supplementary file 1 [file biomolecules-14-01253-s001.zip › biomolecules-3207344-Table S1.pdf]

Table S1. Glucosinolates and non-glucosinolates secondary metabolites content in *ICT2* and *ICS1* relative to WT.

**Glucosinolates**	**ICT2/WT**	**ICS1/WT**
1- // 2- Methylpropyl glucosinolate // Butyl glucosinolate	-0.13358	0.542472
1-Methoxy-3-indolylmethyl glucosinolate	-0.55008	0.059537
2-Hydroxy-2-methylpropyl/4-Hydroxybutyl/ glucosinolate	-1.34267	0.069625
3-//4- Methylpentyl glucosinolate // Hexyl glucosinolate	-0.55498	0.687139
3-Butenyl glucosinolate isomer 2 (Gluconapin)	0.337012	0.58181
3-Indolylmethyl glucosinolate (Glucobrassicin)	-0.01993	0.012969
4-Hydroxy-3-indolylmethyl glucosinolate (4-hydroxy-Glucobrassicin)	-0.11893	0.006655
4-Methylsulfinylbutyl glucosinolate (Glucoraphanin)	0.117602	0.368029
4-Methylthiobutyl glucosinolate (Glucoerucin)	-0.22321	0.320134
5-Methylsulfinylpentyl glucosinolate	-0.02665	0.654665
6-Methylsulfinylhexyl glucosinolate	-0.29634	0.808309
Benzyl glucosinolate (Glucotropaeolin)	-0.2795	0.471744
sulforaphane-glutathione conjugate	0.011604	-0.60385
**Non-Glucosinolates SM**	**ICT2/WT**	**ICS1/WT**
2-Butenoic acid, 2-hydroxy-4-(1-methyl-1H-indol-3-yl)-4-oxo	0.229605	0.412841
3-HYDROXYMETHYLGLUTARATE	-0.17959	0.068421
4-O-methyl-3-demethyl dehydrodiconiferyl alcohol-3,9'-O-diglucoside	0.374754	-0.04705
4-PYRIDOXATE	-0.99037	-0.61508
5-O-Feruloylquinic acid	0.414165	-0.79322
6-Hydroxyindole-3-carboxylate dihexoside	-0.08274	2.055161
6-Hydroxyindole-3-carboxylate hexoside	-0.28894	1.163291
6-Hydroxyindole-3-oyl sinapoyl dihexoside	-0.53844	2.257748
9,10-Dihydrohydroxy jasmonic acid sulfate	-0.21626	1.987668
Acanthoside B	-0.82157	0.597943
Apodanthoside	-0.51976	-0.31727
ARGININE	-0.23775	0.631163
Ascorbic acid	0.092539	-2.92368
AZELATE	-0.59775	0.047673
Caffeoyl dihexose	0.326435	0.025121
Citric acid	0.379102	-0.05829
Dehydroborapetoside B	-1.09725	-1.79982
D-ERYTHROSE	0.130788	0.590351
Dihydroxy methyl benzoic acid hexose	-0.2394	0.217376
Disinapoyl dihexose Isomer 1	0.13654	0.06046
Disinapoyl dihexose Isomer 2	-0.05471	-0.12481
Disinapoyl dihexose Isomer 3	0.07963	0.026465
Disinapoyl hexose Isomer 1	-0.05513	0.395285
Disinapoyl hexose Isomer 2	-0.3189	-0.09078
Disinapoyl hexose Isomer 3	0.398218	0.023133
Disinapoyl hexose Isomer 4	-0.30025	0.387087
Disinapoyl hydroxyferuloyl dihexose Isomer 1	0.85913	-0.05765
Disinapoyl hydroxyferuloyl dihexose Isomer 2	1.217447	-0.12248
Ferulic acid	-0.03463	-1.81266
Feruloyl dihexose Isomer 1	-0.8785	0.412069

Feruloyl dihexose Isomer 2	0.227799	0.397234
Feruloyl dihexose Isomer 3	0.119259	0.098633
Feruloyl glycerol Isomer 1	-0.41609	-0.4834
Feruloyl hexose	0.135366	-0.01909
Feruloyl loganic acid	-0.31315	0.584487
Feruloyl malate	0.193453	-1.10166
Feruloyl sinapoyl hexose	0.314562	1.104523
G(8-5)feruloyl malate	-0.0816	-0.6725
G(8-5)feruloyl malate hexoside isomer 1	0.264771	-0.53194
G(8-5)feruloyl malate hexoside isomer 2	-0.03918	-0.46842
G(8-5)feruloyl malate hexoside isomer 3	-1.66998	0.647381
G(8-O-4)ferulic acid ether hexoside Isomer 1	-0.38004	0.011344
G(8-O-4)ferulic acid ether hexoside Isomer 2	-0.21969	-0.00693
G(8-O-4)ferulic acid ether hexoside Isomer 3	-0.05269	0.069328
G(8-O-4)ferulic acid ether hexoside Isomer 4	-0.61822	0.067781
G(8-O-4)feruloyl malate ether hexoside Isomer 1	-0.14334	-0.59455
G(8-O-4)feruloyl malate ether hexoside Isomer 2	-0.06932	-0.47083
G(8-O-4)feruloyl malate Isomer 1	0.02676	-0.85755
G(8-O-4)feruloyl malate Isomer 2	0.009017	-0.70085
G(8-O-4)G hexoside	-0.26439	0.048084
G(8-O-4)G(8-5)ferulic acid hexoside isomer 1	-0.04734	0.193196
G(8-O-4)G(8-5)ferulic acid hexoside isomer 2	-0.22594	0.127193
G(8-O-4)G(8-5)G hexoside//G(8-O-4)pinoresinol hexoside	0.053779	0.052518
G(8-O-4)G(8-O-4)ferulic acid ether hexoside isomer 1	-0.30743	0.410805
G(8-O-4)G(8-O-4)ferulic acid ether hexoside isomer 2	-0.31537	0.582413
G(8-O-4)G(8-O-4)ferulic acid ether Isomer 1	-0.1552	0.149723
G(8-O-4)G(8-O-4)ferulic acid ether Isomer 2	-0.76531	-0.5259
G(8-O-4)G(8-O-4)ferulic acid ether Isomer 3	-0.07765	0.12467
G(8-O-4)G(8-O-4)G hexoside	0.093951	-0.09313
G(8-O-4)G(8-O-4)sinapic acid ether hexoside Isomer 1	-0.2748	0.354864
G(8-O-4)G(8-O-4)sinapic acid ether hexoside Isomer 2	-0.26182	0.322341
G(8-O-4)lariciresinol hexoside Isomer 1	0.037948	0.022719
G(8-O-4)lariciresinol hexoside Isomer 2	-0.19246	0.653909
G(8-O-4)lariciresinol hexoside Isomer 3	-0.10609	-0.02265
G(8-O-4)S(8-5)ferulic acid hexoside	0.17286	0.377013
G(8-O-4)S(8-8)G dihexoside Isomer 1	-0.17102	-0.15696
G(8-O-4)S(8-8)G dihexoside Isomer 2	-0.28259	0.131234
G(8-O-4)S(8-8)G hexoside Isomer 3	-0.70562	0.00223
G(8-O-4)sinapic acid ester hexoside isomer 1	-0.69671	0.048054
G(8-O-4)sinapic acid ester hexoside isomer 2	-0.74158	0.202344
G(8-O-4)sinapoyl malate ether Isomer 1	-0.32163	-0.45588
G(8-O-4)sinapoyl malate ether Isomer 2	-0.23313	-0.58099
Glucoferulic acid	-0.16792	-0.31205
Glucosyl dihydroascorbigen	-0.09774	0.611103
GLUTAMINE	0.349642	0.169932
Glutathione reduced	-0.02139	-0.16163
Glycosmisic Acid	-0.01675	0.278244
GUANOSINE	0.452796	-0.02569

Hydroxyferulic acid	0.432549	-0.84791
Hydroxyferuloyl hexose Isomer 1	0.954434	-0.05149
Hydroxyferuloyl hexose Isomer 2	1.032572	0.89669
Indole-3-carboxylate dihexoside	-0.23224	1.249751
Indole-3-carboxylate hexose	-0.4673	1.24492
Is dihexoside	-0.07702	-0.25373
Is trihexoside	0.592307	-1.55459
Isoquercetin	0.44473	0.175518
Isorhamnetin 3-O-rutinoside	-0.20104	0.057726
Isorhamnetin-3-O-glucoside	0.15764	-0.00199
Kaempferol	0.691568	0.445106
Kaempferol-3-O/-4'-hexoside	0.036241	0.260372
Kaempferol-3-O-rutinoside	0.237721	0.114611
Kaempferol-7-O-glucoside	0.241261	-0.0212
Km dihexoside	0.552732	0.083425
Km sinapoyl dihexoside	-0.16242	-0.64797
Km trihexoside Isomer 1	2.891323	-2.36168
Km trihexoside Isomer 2	-0.04633	0.195072
Lariciresinol dihexoside	0.242076	-0.14446
Lariciresinol hexoside Isomer 1	-0.15745	-0.11303
Lariciresinol hexoside Isomer 2	-0.27627	-0.06107
Linarin	0.028007	1.50108
Methyl 6-glucosyloxysalicylate	-0.49527	0.273317
METHYLTHIOADENOSINE	-0.19162	0.090096
N-ACETYLGLUTAMATE	-0.44016	0.110588
N-Acetyl-L-tyrosine	0.15479	0.328319
N-ACETYLTRYPTOPHAN	1.410013	4.184388
Naringenin	0.514863	-1.67161
Naringenin chalcone hexose//Naringenin hexose	0.231574	-1.47643
Neoascorbigen	-0.11089	-0.01241
Pantothenic acid (Vitamin B5)	-0.29967	0.133336
p-coumaric acid	0.554355	-0.51734
Phenylalanine	-0.20509	-0.30768
Pinoresinol 4,4'-O-diglucoside	0.16827	0.105528
Protocatechuic acid hexose Isomer 1	-0.23277	1.044264
Protocatechuic acid hexose Isomer 2	0.444983	0.349768
Prunin	0.078302	-1.09559
PYROGLUTAMATE (oxoproline)	0.153527	0.304183
Qn dihexoside	-0.0753	0.105785
Quercetin-3,4'-di-O-glucoside	0.448042	0.216788
Rutin	-0.02704	-0.07103
S(8-O-4)ferulic acid ether hexoside Isomer 1	-0.58288	0.05274
S(8-O-4)ferulic acid ether hexoside Isomer 2	-0.46059	-0.02568
S(8-O-4)ferulic acid ether hexoside Isomer 3	-0.82263	-0.05048
Salicylic acid 2-O-beta-D-glucoside	0.399162	1.593989
Shanzhiside methyl ester	-0.58805	-0.32105
Sinapic acid	-0.34033	-0.18016
Sinapoyl dihexose Isomer 1	-0.45248	0.644347

Sinapoyl dihexose Isomer 2	0.220337	0.542617
Sinapoyl hexose Isomer 1	0.210172	0.028406
Sinapoyl hexose Isomer 2	0.029004	0.224948
Sinapoyl malate Isomer 1	-0.06323	-0.49328
Sinapoyl malate Isomer 2	-0.14692	-0.37243
Sinensin	0.624807	-0.95066
SUBERATE	-0.00547	-0.19642
Symplocosin	-0.25011	-0.17693
THYMIDINE	0.247105	0.155896
Trisinapoyl dihexose	-0.74778	-0.9743
Tryptophan	-0.44757	-0.00371
Uric acid	-0.19889	-1.22226
URIDINE	0.295406	-2.01193
Uridine 5'-monophosphate	-0.17352	0.690825
URIDINE DIPHOSPHATE Hexose	0.060169	0.668808
Vanillic acid	-0.94942	-0.1573
XANTHOSINE	-0.80865	-0.74499
